# Neural substrates in patients with visual-spatial neglect recovering from right-hemispheric stroke

**DOI:** 10.3389/fnins.2022.974653

**Published:** 2022-08-18

**Authors:** Lei Cao, Linlin Ye, Huanxin Xie, Yichen Zhang, Weiqun Song

**Affiliations:** ^1^Department of Rehabilitation, Xuanwu Hospital, Capital Medical University, Beijing, China; ^2^Department of Orthopedics, Beijing Rehabilitation Hospital of Capital Medical University, Beijing, China

**Keywords:** visual-spatial neglect, recovery, dorsal attention network, functional connectivity, ALFF

## Abstract

Visual-spatial attention disorder after stroke seriously affects recovery and quality of life in stroke patients. Previous studies have shown that some patients recovery rapidly from visual-spatial neglect (VSN), but the brain networks underlying this recovery are not well understood. Using functional magnetic resonance imaging, we aimed to identify network differences between patients who rapidly recovered from VSN and those with persistent VSN. The study included 30 patients with VSN who suffered subacute stroke. Patients were examined 2–4 weeks after stroke onset and 4 weeks after the initial assessment. At the last evaluation, patients in the persistent VSN (*n* = 15) and rapid recovery (*n* = 15) groups underwent paper-and-pencil tests. We defined the bilateral frontal eye fields, bilateral intraparietal sulcus in the dorsal attention network, and right temporoparietal junction and ventral frontal cortex areas in the ventral attention network as regions of interest (ROI) and measured whole-brain ROI-based functional connectivity (FC) and amplitude of low-frequency fluctuations (ALFF) in subacute right-hemisphere stroke patients. VSN recovery was associated with changes in the activation of multiple bilateral attentional brain regions. Specifically, persistent VSN was associated with lower FC in the right superior frontal gyrus, right inferior temporal gyrus, right medial orbitofrontal cortex, left precuneus, right inferior parietal gyrus, right medial frontal gyrus, right rectus gyrus, left superior frontal gyrus, left middle cingulate gyrus, right superior temporal pole, right postcentral gyrus, and right posterior cingulate gyrus compared to that in those with rapid recovery, whereas ALFF in the left cerebellum were decreased in patients with persistent VSN. Our results demonstrate that the DAN rather than the VAN, plays a more important role in recovery from VSN, and that the cerebellum is involved in recovery. We believe that our results supplement those of previous studies on recovery from VSN.

## Introduction

Visual-spatial neglect (VSN) is one of the most common cognitive impairments after stroke, particularly in the right hemisphere. VSN occurs in more than half of right hemisphere stroke survivors (Esposito et al., 2020). Unfortunately, in approximately 40% of patients, VSN persists to 1 year after onset (Nijboer et al., 2013) and is associated with poor functional outcome (Chen et al., 2015).

The mechanisms underlying this recovery remain largely unknown. Functional magnetic resonance imaging (fMRI) studies have revealed global activation changes in functional organization far beyond the lesions in VSN patients after stroke (Corbetta et al., 2005). In fact, attentional networks, dorsal attention networks (DAN), and ventral attention networks (VAN) have been identified as crucial components of VSN recovery (Corbetta and Shulman, 2011). A few VSN studies have shown that interhemispheric and intrahemispheric functional connections between attention networks affect recovery from VSN. Baldassarre et al. (2014) highlighted that large-scale changes in network interactions following focal injury are associated with decreased interhemispheric functional connectivity (FC) and the patterns of abnormal functional connectivity between VAN and DAN. Further studies have shown that attention deficit following stroke is significantly more correlated with interhemispheric FC in the DAN and proposed interventions that restore normal patterns of resting FC may be associated with good recovery (Baldassarre et al., 2016). Two important longitudinal studies have demonstrated that improvement of VSN was correlated with an increase in previously depressed interhemispheric FC across attention and network normalization of interhemispheric regions in multiple networks, predominantly the left functional homologs (Ramsey et al., 2016; Umarova et al., 2016).

Recent research extracted the amplitude of low-frequency fluctuations (ALFF) from each region of interest (ROI) as a marker of regional spontaneous neuronal activity in acute right-hemisphere stroke patients with left hemispatial neglect, which can complement the fact that FC analysis cannot offer local dysfunction in a specific area of the network. They also confirmed a reduction in spontaneous neuronal activity in the superior parietal lobule as a consequence of VSN. Intervention at the superior parietal lobule may improve left VSN behavior in stroke patients (Machner et al., 2020).

Previous studies have shown that some patients recover rapidly from VSN (Ye et al., 2020). However, these studies did not consider the effects of the DAN and VAN. We investigated attention network differences between good and poor recovery from VSN to identify specific features of favorable recovery from VSN after a right hemisphere stroke by using two parameters of resting-state fMRI (rs-fMRI): ALFF and FC.

## Patients and methods

### Patient group and clinical details

We retrospectively analyzed data from 30 patients who were admitted to the rehabilitation department of Xuanwu Hospital with a subacute stroke and tested within 2-4 weeks of stroke onset between July 2017 and October 2018. The patients were diagnosed with first-onset right-hemisphere stroke and VSN. The inclusion criteria were: (1) right-handedness, (2) age ≥ 18 years, and (3) availability of informed consent. The exclusion criteria were: (1) non-cooperation with paper-and-pencil tests, (2) previous history of a psychiatric or neurological disorder, (3) claustrophobia, (4) metal implants such as artificial cochlea and cardiac pacemaker; and (5) bilateral-hemisphere or previous brain lesions. All patients received upper- and lower-extremity exercise therapy for 1 h twice daily for 2 weeks. No specific intervention was provided for the VSN. All patients completed paper-and-pencil tests and underwent MRI before exercise therapy.

This study was approved by the local ethics committee and written informed consent was obtained from all participants.

### Neuropsychological assessment

Standardized paper-and-pencil tests were used to evaluate the severity of the VSN. These tests included the line-bisection, line-cancelation, star-cancelation, clock-drawing, and sentence-reading tests. All tests were conducted on a horizontally placed 295 × 210 mm A4 sheet of paper. Behavioral scores were calculated using previously described methods. In the line-bisection task, patients were instructed to mark the midpoints of five horizontal lines of different lengths (between 80 and 160 mm) distributed on the paper. Rightward or leftward deviations from the real midpoint were measured, and a deviation >12% was considered pathological neglect. In the line-cancelation task, patients were instructed to mark 30 line symbols, and a difference between left- and right-sided omissions of three or more was considered pathological neglect. In the star-cancelation task, patients were instructed to mark all small stars that were symmetrically distributed among the distractors on the test paper (27 in the left field, two in the middle field, and 27 in the right field). A difference between left- and right-sided omissions of more than five was considered pathological neglect. In the clock-drawing task, a clock with a random time was presented on paper, and participants were instructed to copy a duplicate clock on the test paper; omission of the left side of the clock compared with the right side was considered pathological neglect. In the sentence-reading task, participants were instructed to read an article aloud in three columns (left, center, and right). Omission of words in one or more sentences was considered pathological neglect. We calculated the scores by considering the total number of targets omitted and left-right differences.

Subjects were diagnosed with VSN when the results of at least two of the paper-and-pencil tests were positive. Behavioral results were independently evaluated by two neurologists.

Paper-and-pencil tests were performed in the early subacute phase (2–4 weeks after stroke) and 4 weeks after the first assessment. MRI was performed in the same day after first assessment. At four weeks after the second assessment, 15 patients still had VSN and were assigned to the persistent VSN group, while the remaining 15 patients were free of VSN, as judged by all behavioral tests, and were assigned to the rapid recovery group. We carefully checked the clinical profiles of all the patients without neglect and found no signs of neglect. Subsequently, a retrospective analysis was performed.

### MRI acquisition

Data were obtained using a GE Signa 3.0T scanner. Foam pads were used to prevent head movement. Functional images were obtained using an echo-planar imaging sequence with the following parameters: 33 axial slices, thickness/gap = 3.5/0 mm, in-plane resolution = 64 × 64, repetition time = 2000 ms, echo time = 30 ms, flip angle = 90°, and field of view = 212 × 212 mm^2^. The resting-state MRI sessions lasted for 8 minutes. Patients were instructed to hold still, plug their ears with sponge earplugs, not think systematically, and not fall asleep. In addition, a T1-weighted sagittal three-dimensional magnetization-prepared rapid gradient echo sequence was acquired using the following parameters:144 slices, repetition time = 2,300 ms, echo time = 3.39 ms, slice thickness = 1 mm, flip angle = 7°, inversion time = 1100 ms, field of view = 200 × 256 mm^2^, and in-plane resolution = 200 × 256. T1-weighted MRI protocols had the following parameters: 32 slices, repetition time = 3823.5 ms, echo time = 24 ms, flip angle = 111°, matrix 256 × 288, 32 slices. The standard slice thickness was 3.0 mm.

### Regions of interest selection

Six ROI were selected based on previous research (Farrant and Uddin, 2015). In the DAN, the ROI were located in the right frontal eye field (rFEF) (Montreal Neurological Institute [MNI] coordinates: *x* = 28, *y* = −8, *z* = 52), left FEF (lFEF) (MNI coordinates: *x* = −28, *y* = −8, *z* = 52), right intraparietal sulcus (rIPS) (MNI coordinates: *x* = 21, *y* = −58, *z* = 53), left IPS (lIPS) (MNI coordinates: *x* = −28, *y* = −56, *z* = 44), right temporoparietal junction (TPJ) (MNI coordinates: *x* = 60, *y* = −48, *z* = 22), and right ventral frontal cortex (VFC) (MNI coordinates: *x* = 42, *y* = 20, *z* = −6) for ventral attention networks.

### Imaging data analysis

The fMRI data were processed and analyzed performed using the Data Processing Assistant of the rs-fMRI software (DPARSF Advanced Edition V5.2)^1^. The first 10 volumes for each participant were discarded. All images were time-shifted, such that the slices were temporally aligned. The images were then realigned, and all participants moved no more than 3 mm in translational or 3° in rotational dimensions. The images were then co-registered with anatomical images, which were segmented into gray and white matter. Anatomical images were obtained using Diffeomorphic Anatomical Registration Exponentiated Lie Algebra (DARTEL). First, a sample-specific template was generated from the T1-weighted images. Second, the individual anatomical images were normalized non-linearly to the template, followed by linear registration to the MNI template. The images were smoothed using a Gaussian filter with a full width at half maximum of 4 mm. Finally, nuisance signals, including 24 head-motion parameters, cerebrospinal fluid signals, and white matter signals, were regressed from the MRI data.

Further preprocessing, including removal of linear trends, temporal band-pass filtering (0.01–0.1 Hz), and ALFF, was performed using the Data Processing Assistant of rs-fMRI software (DPARSF Advanced Edition V5.2; See Text Footnote 1). All ALFF map results were converted into z-maps.

To perform the FC analyses, time series from the resting-state scan were extracted for the subject-specific ROI in the rFEF, lFEF, rIPS, and lIPS for DAN, and right TPJ and right VFC for VAN by averaging the time series of all voxels in the spherical ROI (radius = 6 mm). Whole-brain voxel-based FC analysis was used to calculate the FC strength between each voxel in each subject and the ROI and convert it into z-maps.

### Statistical analyses

SPSS (version 22.0; IBM Corp., Armonk, NY, United States) was used for all statistical analyses. Demographic characteristics, including symptom severity, were analyzed using descriptive statistics (mean differences ± standard deviation (x ± s) for each group. Sex and type of stroke were compared using the χ2 test, and other quantitative variables were compared using the independent-sample *t* test. Statistical significance was set at *p* ≤ 0.05.

Group-level statistical analyses were performed using the statistical analysis model in DPARSF. A two-sample t-test was conducted on the individual z-ALFF and z-ROI FC maps of the two groups with a small volume correction for the one-sample result masks. Multiple-comparison corrections were performed using the Gaussian random field correction. Significant between-group differences met the criteria of corrected *p* < 0.01 for voxel level and *p* < 0.05 for cluster size level.

## Results

### Patient classification and demographics

**FIGURE 1:**
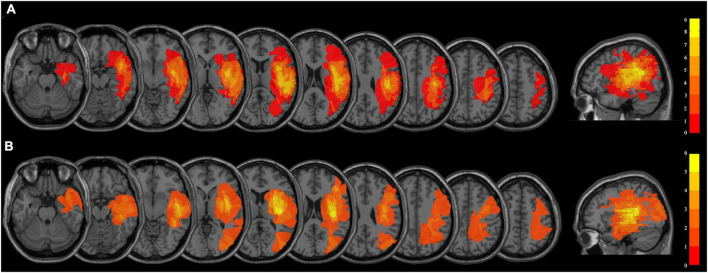
**(A)** Overlapped lesion plots of the patients with persistent VSN. **(B)** Overlapped lesion plots of the patients with rapid recovery VSN.

The average age of the persistent VSN patients and the rapid recovery patients were 57.27 ± 10.93 and 56.20 ± 10.93 years, respectively (the persistent VSN group: three women, 12 men, age 32–73 years; the rapid recovery group: four women, 11 men, age 37–71 years). There were no significant differences in age, sex, type of stroke, years of education, initial paper-and-pencil test scores, or clinical course following stroke between the two groups (*p* > 0.05). The demographic and clinical characteristics of the patients are presented in Table 1. The overlapped lesion plots of the patients are presented in Figure 1.

**TABLE 1:** Demographic data and clinical data.

Patient ID	Age	Sex	Time science stroke (days)	Type of stroke	Years of education	Line bisection (deviation%)	Line cancelation (all omissions)	Star cancelation (all omissions)	Clock drawing	Sentence reading omissions
P1	32	M	28	CH	18	54.8	28	38	+	5
P2	69	M	28	CI	15	39.7	15	38	+	4
P3	54	M	25	CI	12	35.66	26	48	+	9
P4	51	M	20	CI	14	64.95	8	36	+	6
P5	57	M	21	CI	11	17.26	5	14	+	2
P6	51	M	27	CI	12	14.72	6	24	+	2
P7	57	F	20	CH	16	78.04	24	48	+	13
P8	70	M	20	CI	9	52.95	9	13	+	2
P9	62	M	14	CI	9	69.97	17	48	+	7
P10	55	M	19	CI	15	20.95	6	42	+	4
P11	41	F	21	CI	18	21.46	6	26	+	4
P12	73	M	23	CI	8	82.69	15	27	+	9
P13	65	F	27	CI	12	42.37	4	22	+	2
P14	64	M	24	CI	9	50	23	46	+	7
P15	58	M	14	CI	12	32.75	5	9	+	2
R1	37	F	26	CI	18	69.46	21	48	+	4
R2	53	M	25	CH	12	42.34	14	46	+	7
R3	71	M	26	CI	6	56.07	18	34	+	4
R4	50	M	21	CI	3	69.96	6	25	+	6
R5	65	M	19	CI	9	33	15	40	+	5
R6	47	M	22	CI	12	25.4	14	25	+	2
R7	42	M	22	CI	16	68.37	12	30	+	6
R8	52	M	17	CI	15	38.26	4	25	+	4
R9	52	M	27	CI	14	62.96	10	45	+	13
R10	47	F	18	CI	19	28.7	19	34	+	2
R11	60	M	26	CI	9	75.23	10	17	+	6
R12	67	M	26	CH	12	42.27	7	33	+	4
R13	72	F	20	CI	6	20.2	6	16	+	2
R14	59	F	21	CI	12	16.67	9	44	+	4
R15	69	M	20	CI	9	30	5	14	+	2

P1-P15: patients with persistent VSN; R1-R15: patients with rapid recovery VSN; M: male; F: female; CH: cerebral hemorrhage; CI: cerebral ischemia.

### Neuropsychological performance

**FIGURE 2:**
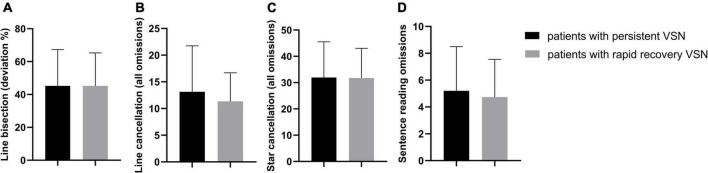
The behavioral scores in two groups, all *p* > 0.05. **(A)** Line bisection (deviation %); **(B)** Line cancellation (all omissions); **(C)** Star cancellation (all omissions); **(D)** Sentence reading omissions.

The behavioral scores in the early subacute phase showed no significant difference between the rapid recovery and persistent VSN patients (Line bisection *t* = −0.005, *p* = 0.996; Line cancelation *t* = 0.688, *p* = 0.498; Star cancelation *t* = 0.044, *p* = 0.965; Sentence reading *t* = 0.417, *p* = 0.680). The behavioral scores of the two groups are presented in Figure 2.

### Differences in whole-brain functional connectivity of dorsal attention networks nodes

**FIGURE 3:**
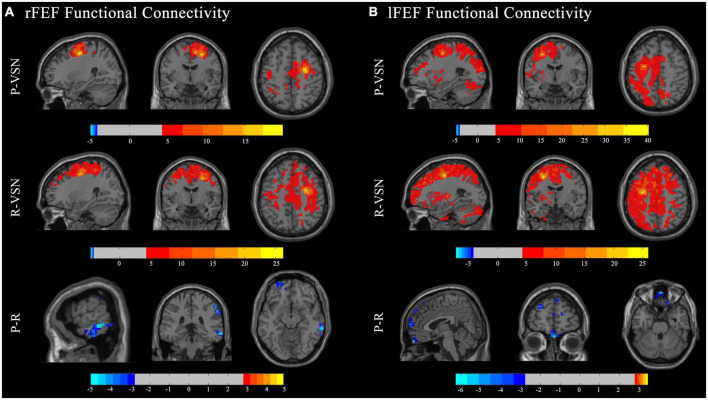
Functional connectivity (FC) maps. Individual and group comparisons of resting state functional connectivity of seed ROIs rFEF **(A)** and lFEF **(B)**. First panel show individual one sample *t*-test for persistent VSN(P-VSN) (*p* < 0.001). Second panel show individual one sample *t*-test for rapid recovery VSN(R-VSN) (*p* < 0.001). Remaining panels show two-sample t-tests comparing persistent VSN with rapid recovery VSN(P-R) (*p* < 0.01).

In the persistent VSN group, the rFEF showed strong FC with the right precentral gyrus, left precuneus, left precentral gyrus, left middle cingulate gyrus, left supplementary motor area, and left cerebellum. In the rapid recovery group, the rFEF showed strong FC with the right supplementary motor area, right superior frontal gyrus, right precentral gyrus, left lingual gyrus, left cerebellum, left medial orbitofrontal cortex, right middle temporal gyrus, and left middle frontal gyrus (Figure 3A).

In the persistent VSN group, the lFEF showed strong FC with the left superior frontal gyrus, left triangular part of the inferior frontal gyrus, and left putamen. In the rapid recovery group, the lFEF showed strong FC with the left precentral gyrus, right cerebellum, left inferior temporal gyrus, and left cerebellum (Figure 3B).

**FIGURE 4:**
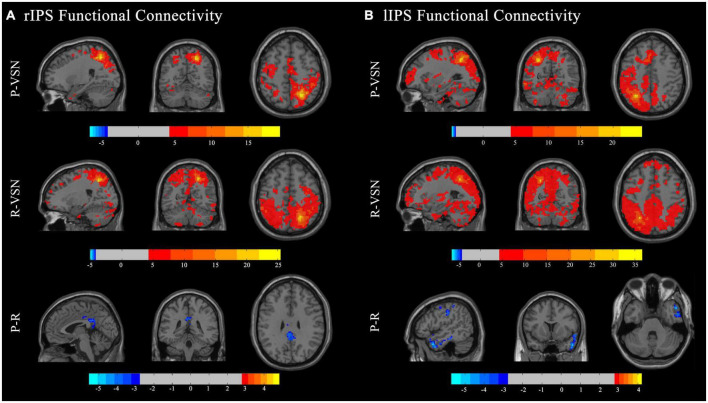
Functional connectivity (FC) maps. Individual and group comparisons of resting state functional connectivity of seed ROIs rIPS **(A)** and lIPS **(B)**. First panel show individual one sample t-test for persistent VSN(P-VSN) (*p* < 0.001). Second panel show individual one sample t-test for rapid recovery VSN(R-VSN) (*p* < 0.001). Remaining panels show two-sample t-tests comparing persistent VSN with rapid recovery VSN(P-R) (*p* < 0.01).

In the persistent VSN group, the rIPS showed strong FC with the right superior parietal gyrus, left lingual gyrus, left superior marginal gyrus, right supplementary motor area, left middle cingulate gyrus, right middle temporal pole, right superior marginal gyrus, and right fusiform gyrus. In the rapid recovery group, the rIPS showed strong FC with the right superior parietal gyrus, left thalamus, right superior frontal gyrus, right middle frontal gyrus, left medial superior frontal gyrus, left cerebellum, right cerebellum, right middle temporal gyrus, right superior frontal gyrus, left triangular part of the inferior frontal gyrus, left middle temporal gyrus, and left opercular part of the inferior frontal gyrus (Figure 4A).

In the persistent VSN group, the lIPS showed strong FC with the left superior parietal gyrus and left cerebellum. In the rapid recovery group, the lIPS showed strong FC with the left inferior parietal gyrus and right rolandic operculum (Figure 4B).

The FC between the DAN and voxel-based whole-brain of the rapid recovery group was significantly increased compared to that of the persistent VSN group (*p* < 0.01). The FC between the rFEF, lFEF, rIPS, lIPS, and the whole brain is shown in Figure 3. The rFEF ROI showed significantly stronger FC with the right superior frontal gyrus, right inferior temporal gyrus, right medial orbitofrontal cortex, left precuneus, and right inferior parietal gyrus in the rapid recovery group than in the persistent VSN group. The lFEF ROI showed significantly stronger FC with the right superior frontal gyrus, right medial frontal gyrus, right rectus gyrus, and left superior frontal gyrus in the rapid recovery group than in the persistent VSN group. The rIPS ROI showed significantly stronger FC with the left middle cingulate gyrus in the rapid recovery group than in the persistent VSN group. The lIPS ROI showed significantly stronger FC with the right superior temporal pole, right postcentral gyrus, and right posterior cingulate gyrus in the rapid recovery group than in the persistent VSN group (Table 2 and Figures 3, 4).

**TABLE 2:** Group differences in functional connectivity of DAN and VAN ROIs.

Seed	Functional connectivity	MNI coordinates			*Z*-score	Cluster size
		
		*X*	*Y*	*Z*		(voxels)
rFEF	right superior frontal gyrus	15	48	33	−4.92	456
	right inferior temporal gyrus	66	−36	−12	−5.01	211
	right medial orbitofrontal cortex	−15	69	−3	−4.59	186
	left precuneus	−6	−66	39	−3.55	152
	right inferior parietal gyrus	60	−33	39	−4.87	114
lFEF	right superior frontal gyrus	6	54	39	−4.52	206
	right medial frontal gyrus	−21	60	27	−5.36	204
	right rectus gyrus	3	57	−27	−5.53	116
	left superior frontal gyrus	−9	42	54	−4.45	110
rIPS	left middle cingulate gyrus	−3	39	30	−3.94	104
lIPS	right superior temporal pole	51	12	−33	−5.72	428
	right postcentral gyrus	54	21	39	−4.53	376
	right posterior cingulate gyrus	0	−54	24	−4.13	117

### Differences in whole-brain functional connectivity of ventral attention networks nodes

In both the persistent VSN and rapid recovery groups, the right TPJ showed strong FC in the right superior temporal gyrus.

In both the persistent VSN and rapid recovery groups, the right VFC showed strong FC in the right insula.

In terms of FC with other VAN regions, no group differences were found in VAN (TPJ/VFC) connections. In other words, the persistent VSN and rapid recovery groups did not differ in FC strength between VAN and whole-brain voxel-based functional connections.

### Amplitude of low-frequency fluctuations

**FIGURE 5:**
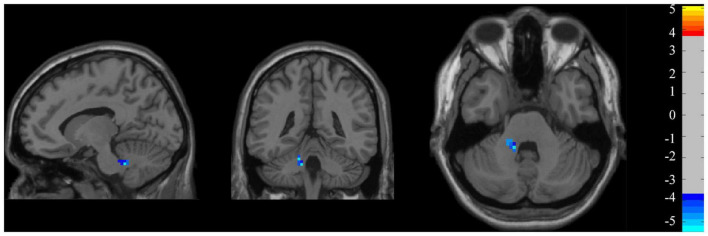
ALFF maps. ALFF maps show two-sample *t*-tests comparing persistent VSN with rapid recovery VSN(P-R) (*p* < 0.001).

At the group level (Figure 5), the z-ALFF value did not differ significantly between the persistent VSN and rapid recovery groups for most ROI. Only the mean z-ALFF value in the left cerebellum anterior lobe was significantly reduced in the persistent VSN group compared with that in the rapid recovery group.

## Discussion

VSN is common following stroke, but the neural substrates for recovery are unclear. Our results showed that the interhemispheric and intrahemispheric FC of the DAN to the whole brain was reduced in patients with persistent VSN compared to that in patients with rapid recovery. The results provide novel insights into the mechanisms underlying recovery; that is, the FC of the DAN, but not the VAN, key nodes play a crucial role in recovery from VSN. In addition, local reduction in spontaneous neuronal activity in the cerebellum is associated with poor recovery.

Recovery is associated with changes in FC in the DAN. Consistent with previous research, changes in interhemispheric FC are sensitive markers related to recovery, especially in the DAN (Ramsey et al., 2016; Umarova et al., 2016). In this study, we found that the ipsilesional DAN was more related to recovery by an increase in the interhemispheric FC of the key nodes in the ipsilesional frontal, parietal, and temporal regions, whereas the contralesional DAN was more related to the key nodes of the lesionlateral hemisphere. This indicates that both the ipsilesional and contralesional hemispheres are involved in compensation of the attention network after injury. This may involve reorganization and integration of the attention network. Umarova et al. conducted a longitudinal study on patients with VSN and found that transneuronal changes in the contralesional frontoparietal and bilateral occipital connections were distinctly related to an unrecovered VSN in patients with chronic stroke. This revealed a large-scale structural reorganization of the visual-spatial attention network after stroke, especially in the trans-hemisphere (Umarova et al., 2017). Lunven et al. believed that persistent neglect is due to disconnection between the DAN and VAN. This emphasizes the importance of network-based integration (Lunven et al., 2015).

Large-scale reorganization of functional connections in the whole-brain network structure has been previously reported. Animal studies have shown that when one hemisphere is damaged, activation of the entire attention network decreases, in addition to the decrease in activation of the ipsilesional attention network. Synaptic loss occurred 2–4 days after stroke, the normal network activation mode was disrupted, and the entire attention network was in an overexcited state 7 days after stroke, and characterized by the expansion of excitatory neurites and an increase in synapses. This phenomenon exists not only around the lesion but also in the contralesional hemisphere (Deuel and Collins, 1983; Gonzalez et al., 2004). A recent study found that axonal and retrograde degeneration occurs not only occur around the lesion but also in distant regions that are not directly connected to the lesion by transneuronal remodeling (Fornito et al., 2015). In addition, the lesions in our patients were located within the territory of the right middle cerebral artery. According to some studies, hemispheric differences occur in different hemispheres after an acute middle cerebral artery infarction. Compensatory activation occurs in related brain areas of the dominant hemisphere after acute infarction in the middle cerebral artery of the non-dominant hemisphere (Gao et al., 2021).

In the current study, we also found that patients with persistent VSN showed weaker FC of the DAN to the whole brain, particularly in the frontal region, as revealed by group differences in the whole-brain FC of key DAN nodes. Previous studies have indicated an important role of the frontal lobe in the DAN. The contralesional prefrontal cortex supports to residual ipsilesional attention centers to accelerate restoration or the reorganization of the perilesional cortex (Umarova et al., 2016). According to the degree of injury to the patient, the prefrontal cortex produces different advanced controls over movement and sensation systems (Liu et al., 2016). In stroke patients with mild dysfunction, rs-fMRI has shown that a stronger FC between the prefrontal lobe and motor cortex is associated with better recovery (Lam et al., 2018). However, in patients with severe dysfunction after stroke, the prefrontal cortex may play a greater role in functional recovery, and FC between the prefrontal sensorimotor cortex was significantly correlated with recovery (Yin et al., 2012). In unilateral hemispheric prefrontal lobe injury in macaque monkeys, FC between the contralesional prefrontal cortex and ipsilesional parietal cortex was related to recovery (Adam et al., 2020). Activation of the contralesional prefrontal cortex is a compensatory strategy underlying cortical mechanisms during the recovery process after VSN (Takamura et al., 2016).

In addition to conventional FC analyses, we extracted the ALFF from each ROI as a marker of spontaneous regional neuronal activity. The ALFF is a low-frequency fluctuation index that improves sensitivity and specificity in the detection of spontaneous brain activitiy (Zou et al., 2008). Interestingly, we found that the ALFF values in the left cerebellum anterior lobe were lower in the persistent VSN group than in the rapid recovery group. This indicates that the cerebellum has a positive effect on recovery and may compensate for the VSN by affecting the attention network. Inconsistent with previous studies by Machner et al., we found that the ALFF of fMRI in the cerebellum of patients with persistent VSN was lower than that of patients with rapid recovery. However, the reduced ALFF also reflects local disruption of long-range neuronal network communication in VSN patients (Machner et al., 2020).

These findings do not contradict the previous proposal of a crucial role for the DAN in VSN. However, the functional role of cerebellar structures in cognitive networks remains poorly understood. In fact, an article published in *Science* in 1997 was the first fMRI study propose the role of the cerebellum in attention processing (Allen et al., 1997). However, in the following 20 years, the role of the cerebellum in motor function gradually came into view, and its role in cognition was rarely mentioned. Buckner et al. identified a region spanning cerebellar lobules VIIb and VIIIa that exhibited connectivity with the cortical DAN (Buckner et al., 2011). In electrophysiological tests of patients with cerebellar injury, the lateral CrusI and CrusII of the cerebellum were associated with the attention network of the frontal parietal lobe (Striemer et al., 2015). A few months later, Brissenden et al. found that a region located in the posterior cerebellar lobe (lobules VIIb and VIIIa) showed strong activation under the attention multi-objective paradigm through visual attention-related tasks, which provided evidence that the DAN extended functionally to the cerebellum (Brissenden et al., 2016). Subsequently, this team demonstrated that neurons in the VIIb/VIIIa area of the cerebellum generates stronger recruitment during spatial coding tasks and were strongly activated under visual working memory load. This performance was mirrored by the corresponding areas of the cerebral hemisphere, which further proved that there was a highly specific cortical cerebellar network involved in attention function (Brissenden et al., 2018). Furthermore, a recent study showed that the cerebellum, as an attention structure across the cortex and subcortex, was strongly activated during the attention paradigm, which provides new evidence for the cerebellum as an important node of the DAN (Brissenden et al., 2016). There are more neurons in the intact cerebellum than in the cerebral cortex that may participate in the DAN and compensate for recovery through the cerebellum-to-cortex circuit. Consistent with our results, all the patients with good outcomes showed cerebellar activation (Lasek-Bal et al., 2018). The results of these studies also support the idea that neurological deficits are compensated by engagement of larger areas of the cerebral cortex.

The potential limitations of this study should be considered. First, this was a retrospective study that compared differences in FC between patients with different outcomes. However, we did not include right brain lesions without VSN. Second, due to the high requirements of rs-fMRI for patients, we included only patients who could cooperate with the examination. Therefore, we were unable to assess the attention network of patients with a more serious disability who could not cooperate in the examination. Third, our study focused on patients with VSN in the early subacute stage after stroke. There has been no follow-up of these patients, and it is unknown whether patients with persistent VSN also experience neglect in the chronic phase. Finally, patients with cerebral hemorrhage and cerebral infarction were included in this study. To minimize the impact of cerebral hemorrhage on the results, we included examinations of patients 2–4 weeks after stroke. Cerebral hemorrhage was resolved in four patients, and there was no obvious perilesional edema. We also believe that it would be better to unify the types of stroke.

## Conclusion

Persistent VSN patients with stroke show interhemispheric and intrahemispheric FC of the DAN to the whole brain, and the ALFF value in the left cerebellum anterior lobe was reduced compared to that in those with rapid recovery. Our results demonstrate that the DAN rather than the VAN, plays a more important role in recovery from VSN, and that the cerebellum is involved in recovery. Our findings provide activation in DAN between key notes as important evidence for recovery and facilitate the design of therapeutic approaches such as non-invasive brain stimulation to ensure better recovery of VSN and development of newer interventions after stroke.

## Data availability statement

The original contributions presented in this study are included in the article/supplementary material, further inquiries can be directed to the corresponding authors.

## Ethics statement

The studies involving human participants were reviewed and approved by Ethics committee of Xuanwu Hospital. The patients/participants provided their written informed consent to participate in this study.

## Author contributions

LC: drafting and revising the manuscript, study concept and design, and analysis and interpretation of data. LY: study concept and design, acquisition of data, revising the manuscript, and obtaining funds. HX: analysis and interpretation of data. YZ: analysis of data and acquisition of MRI data. WS: study concept and design, analysis and interpretation of data, and study supervision. All authors contributed to the article and approved the submitted version.

## Footnotes

1
http://www.rfmri.org/DPARSF
